# VHL Type 2B Mutations Retain VBC Complex Form and Function

**DOI:** 10.1371/journal.pone.0003801

**Published:** 2008-11-25

**Authors:** Kathryn E. Hacker, Caroline Martz Lee, W. Kimryn Rathmell

**Affiliations:** Department of Medicine, Curriculum in Genetics and Molecular Biology, Lineberger Comprehensive Cancer Center, University of North Carolina at Chapel Hill, Durham, North Carolina, United States of America; East Carolina University, United States of America

## Abstract

**Background:**

von Hippel-Lindau disease is characterized by a spectrum of hypervascular tumors, including renal cell carcinoma, hemangioblastoma, and pheochromocytoma, which occur with *VHL* genotype-specific differences in penetrance. *VHL* loss causes a failure to regulate the hypoxia inducible factors (HIF-1α and HIF-2α), resulting in accumulation of both factors to high levels. Although HIF dysregulation is critical to VHL disease-associated renal tumorigenesis, increasing evidence points toward gradations of HIF dysregulation contributing to the degree of predisposition to renal cell carcinoma and other manifestations of the disease.

**Methodology/Principal Findings:**

This investigation examined the ability of disease-specific *VHL* missense mutations to support the assembly of the VBC complex and to promote the ubiquitylation of HIF. Our interaction analysis supported previous observations that VHL Type 2B mutations disrupt the interaction between pVHL and Elongin C but maintain partial regulation of HIF. We additionally demonstrated that Type 2B mutant pVHL forms a remnant VBC complex containing the active members ROC1 and Cullin-2 which retains the ability to ubiquitylate HIF-1α.

**Conclusions:**

Our results suggest that subtypes of *VHL* mutations support an intermediate level of HIF regulation via a remnant VBC complex. These findings provide a mechanism for the graded HIF dysregulation and genetic predisposition for cancer development in VHL disease.

## Introduction

von Hippel-Lindau (VHL) disease is an autosomal dominant familial cancer syndrome caused by germline mutation or loss of the *VHL* tumor suppressor gene that affects approximately 1 in 36,000 individuals [1]. Individuals with VHL disease develop an array of tumors, including clear cell renal cell carcinomas (ccRCC), cerebellar and retinal hemangioblastomas, and pheochromocytomas [1]. VHL disease is divided by genotype into subtypes which predict the spectrum of risk for development of *VHL*-associated lesions [2]–[4]. Type 1 VHL disease predisposes to the development of ccRCC and hemangioblastoma. All patients with Type 2 VHL disease are at risk for pheochromocytoma. Type 2A VHL disease is further characterized by high risk for hemangioblastoma, and Type 2B VHL disease is associated with high risk for both hemangioblastoma and RCC. Type 2C VHL disease individuals exclusively develop pheochromocytoma [5]. Patients homozygous for the Arg200Trp (R200W) *VHL* mutation, located in the extreme C-terminal domain of the 213 amino acid VHL protein (pVHL), develop Chuvash Polycythemia [6], [7]. Biallelic inactivation of pVHL has also been reported in upwards of 90% of individuals with sporadic ccRCC [8]. Thus, a thorough understanding of wild-type and disease-associated mutant pVHL activities has potential to impact a broad spectrum of affected patients [1], [9], [10].

The VHL protein acts as the substrate recognition subunit of an E3 ubiquitin ligase complex analogous in structure to the SCF complex. The SCF and SCF-like complexes typically contain four subunits, including a RING finger protein (ROC1/Rbx1), a cullin protein (CUL/Cul), and two adaptor proteins linking the cullin to the substrate binding protein [11]. In the pVHL E3 complex (VBC), pVHL acts as the substrate binding protein and is responsible for the specificity of the complex-target interaction [12]. Human pVHL directly interacts with Elongin C, while Elongin B links pVHL-Elongin C to cullin 2 (CUL2)-ROC1 [13]–[15]. The VBC complex serves as a platform through which the E2 ubiquitin-conjugating enzyme, bound by CUL2-ROC1, and the pVHL-bound substrate are brought into proper positioning for ubiquitin transfer [11]. In the VBC complex, ROC1 functions to recruit the E2 enzyme and also promotes internal complex stability [12], [14].

The primary targets of the VBC complex are the hypoxia inducible factors, HIF-1α and HIF-2α. The HIF factors direct the transcriptional response to hypoxia by activating the expression of genes involved in angiogenesis, cell proliferation, erythropoiesis, energy metabolism, and apoptosis [16]–[21].

Loss of the pVHL tumor suppressor, as occurs with Type 1 *VHL* mutations, is believed to promote renal tumorigenesis primarily through loss of pVHL-mediated HIF regulation [22]. Correspondingly, Type 2C *VHL* missense mutations display fully intact regulation of HIF factors, consistent with the lack of conveyed risk for RCC development [23]. In the case of Type 2A and Type 2B *VHL* missense mutations, titrated degrees of HIF regulation appear to correlate with the subtype-specific risk of ccRCC [21], [24]. While the differing capabilities of VHL disease-associated mutants to regulate HIF have been explored [20], [21], [23]–[25], the mechanism of retained HIF regulation and the link between differing levels of HIF regulation and the clinical spectrum observed in VHL disease is not yet fully understood. In this study, we re-examined the effect of *VHL* missense mutations to disrupt the formation of the VBC complex, and demonstrate that characteristic Type 2B *VHL* mutations form a low-abundance VBC complex which retains the ability to ubiquitylate HIF-1α.

## Materials and Methods

### Cell Lines

*Vhl*-null embryonic stem (ES) cells or *VHL*-deficient 786-0 RCC cells were transfected with vectors specifying human wild-type *VHL* or representative *VHL* mutants Y112H (Type 2A), R167Q (Type 2B), L188V (Type 2C), or R200W (Chuvash polycythemia) using vectors and techniques as previously described [25]. 786-0 cells expressing the *VHL* mutant D121G (Type 2B), generated as previously described [26], were generously provided by Dr. William Kim, Chapel Hill, NC. *Vhl*-null murine ES cells and transfected derivatives were maintained in culture media comprised of Dulbecco's Modified Eagle Medium (DMEM, various manufacturers), supplemented with 10% ES cell-certified fetal bovine serum (Invitrogen, Carlsbad, CA), non-essential amino acids, L-glutamine, 2-mercaptoethanol, and leukemia inhibitory factor, and were grown on gelatin-coated plates in the absence of feeder cells. Renal cell carcinoma 786-0 cells were acquired from the American Type Culture Collection (Manassas, VA). 786-0 cells and transfected derivatives were maintained in DMEM, supplemented with 10% FBS, non-essential amino acids, L-glutamine, and 2-mercaptoethanol. All cultures were maintained at 37°C in 5% CO_2_. For hypoxia mimetic experiments, cells in log-phase growth were placed in media supplemented with 100 mM cobalt chloride (Sigma, St. Louis, MO) or fresh unsupplemented media.

### Immunoblot Analysis

Cells were lysed in Mammalian Protein Extraction Reagent (M-PER; Pierce Biotechnology, Rockford, IL) supplemented with Complete Mini Protease Inhibitor Cocktail (Roche, Basel, Switzerland). The Bradford Quantification Method (Amresco, Solon, OH) was used to determine protein concentration. Cell lysates were resolved by SDS-PAGE and subsequently transferred to Hybond ECL nitrocellulose membrane (GE Healthcare, United Kingdom). Immediately following transfer, membranes were stained with Ponceau S to confirm even transfer, blocked in 5% nonfat dry milk diluted in phosphate-buffered saline containing 0.1% Tween-20 (PBS-T), and then probed with the following primary antibodies: rabbit polyclonal anti-HA tag (Abcam, Cambridge, MA: ab9110, 1∶1000), mouse monoclonal anti-pVHL (Abcam, ab11189, 1∶2000), rabbit polyclonal anti-cullin-2 (Abcam: ab1870, 1∶1000), rabbit polyclonal anti-ROC1 (Abcam: ab2977, 1∶500), goat polyclonal anti-Elongin C (Santa Cruz, Santa Cruz, CA: sc-1559, 1∶200), rabbit polyclonal anti-Elongin B (Santa Cruz: sc-11447, 1∶200), mouse monoclonal anti-Myc tag (Cell Signaling, Danvers, MA: 9B11, 1∶1000), mouse monoclonal anti-HIF-2α (GeneTex, San Antonio, TX: GTX30123, 1∶1000), and rabbit polyclonal anti-Ku80 (GeneTex: GTX70485, 1∶2000). Secondary antibodies used were anti-mouse, anti-rabbit, and anti-goat IgG conjugated to horseradish peroxidase (various manufacturers) and detected with the ECL Plus Western Blotting System (GE Healthcare) using exposure to BlueLite autoradiography film (ISC BioExpress, Kaysville, UT) and processing via a Kodak RP X-OMAT Processor (Rochester, NY).

### Immunoprecipitation Analysis

M-PER cell lysates were subjected to immunoprecipitation (IP) using either the Profound Mammalian HA Tag IP/Co IP Kit or the Profound Mammalian Myc Tag IP/Co IP Kit, as per manufacturer's specifications (Pierce Biotechnology). For the reverse co-IP analysis, stably-transfected 786-0 cell lines were transiently transfected with a plasmid encoding myc-tagged cullin-2, a generous gift from Dr. Y. Xiong, Chapel Hill, NC, using Solution V of the Amaxa Tranfection System (Amaxa, Gaithersberg, MD). Twenty hours post-transfection, transfected cells were incubated in media supplemented with 5 µM MG-132 (Calbiochem, Gibbstown, NJ) proteasome inhibitor or fresh unsupplemented media for four hours, followed by protein extraction with M-PER and IP analysis as above.

### *In vitro* HIF-1α Ubiquitylation Assay

An *in vitro* ubiquitylation assay was adapted from the protocol developed by Cockman et al. [18]. 786-0 cell lines incubated for four hours in 5 µM MG-132 were washed and collected in PBS. The cells were then washed twice in Ub Extraction Buffer (20 mM Tris, pH 7.5, 5 mM Kcl, 1.5 mM MgCl_2_, 1 mM DTT) and disrupted using a dounce homogenizer. The cell lysates were centrifuged at 10,000×g for 10 minutes at 4°C. Each reaction was set up in a total volume of 40 µL, containing 23 µL cell extract, 5 µL HIF1α-myc substrate, and 12 µL reaction solution. The “reaction solution” was composed of ATP Regenerating System (20 mM Tris, pH 7.5, 10 mM ATP (GE Healthcare)), 10 mM magnesium acetate (Promega Corporation, Madison, WI), 300 mM creatine phosphate, 0.5 mg/mL creatine phosphokinase (MP Biomedicals, LLC, Irvine, California), 20 µg ubiquitin, and 150 uM ubiquitin aldehyde (Biomol International, Plymouth Meeting, PA). When reactions excluded a specific component, nuclease-free dH_2_O was substituted to maintain the total reaction volume. Reactions were incubated at 30°C for 270 minutes and then subjected to immunoblot analysis. HIF1α-myc substrate was produced through TNT® coupled Wheat Germ Extract Systems (Promega, Madison, WI) from a plasmid encoding full-length functional human HIF1α-myc protein, a generous gift from Dr. M. C. Simon, Philadelphia, PA.

## Results

### Type 2B mutant pVHL proteins promote incomplete normoxic HIF-2α stabilization

**Figure 1:**
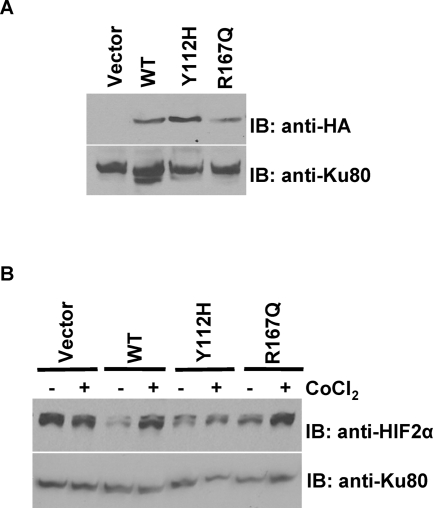
VHL disease associated mutations demonstrate a graded amount of HIF Regulation. A. Anti-HA immunoblot for expression of HA-tagged human pVHL in transgenic 786-0 clones. Whole-cell protein extracts were prepared from 786-0 clones deficient for *VHL* expression (Vector) or modestly expressing wild-type (WT) or missense (Y112H, R167Q) mutant HA-tagged human pVHL. B. Anti-HIF-2α immunoblot for HIF stabilization in 786-0 clones. Whole-cell protein extracts were prepared from *VHL*-deficient 786-0 cells or WT or mutant HA-pVHL-rescued 786-0 cells incubated in the presence or absence of the hypoxia mimetic CoCl_2_ for 24 hours. Ku80 immunoblot was used as a control for equal loading.

We have previously shown that human *VHL* mutations representative of Types 2A and 2B VHL disease impart an intermediate degree of HIF-2α regulation in a *Vhl*-null murine ES cell expression system [25]. In order to evaluate this trend in human RCC cells, 786-0 RCC-derived cells, known to lack pVHL expression and over-express HIF-2α, were reconstituted with expression vectors encoding wild-type or VHL disease-specific mutant *VHL* cDNA. Individual clones expressing mutant pVHL comparable to wild-type levels were selected for subsequent experiments. Figure 1A depicts a representative immunoblot for the expression of wild-type as well as RCC-associated Type 2A (Y112H) and 2B (R167Q) mutant HA-tagged pVHL in 786-0 RCC clones.

To study the ability of RCC-associated mutant pVHL to regulate HIF in 786-0 cells, the hypoxia mimetic cobalt chloride (CoCl_2_) was used to simulate hypoxic conditions. Cells were placed in either standard growth media or media supplemented with 100 mM CoCl_2_for 24 hours followed by analysis of HIF-2α protein levels by immunoblot (Figure 1B). *VHL*-null vector-only transfected cells (vector) failed to suppress HIF-2α under standard conditions and lacked further induction under simulated hypoxic conditions. Introduction of wild-type HA-pVHL restored normoxic suppression and CoCl_2_ induction of HIF-2α. Type 2A *VHL* mutant Y112H cells failed to completely suppress HIF-2α levels, as expected due to the predicted disruption of the HIF interaction domain [15]. Type 2B *VHL* mutant R167Q cells displayed partial suppression of HIF-2α while retaining HIF-2α stabilization in response to CoCl_2_. These observations were confirmed in multiple independently-derived 786-0 clones (data not shown).

### Type 2B mutant protein participates in a VBC complex containing Cullin2

To determine the correspondence between the observed partial retention of HIF-2α regulation and formation of a competent VBC complex, we analyzed the interaction of wild-type and disease-specific mutant HA-pVHL with known components of the VBC complex by co-IP and reverse co-IP studies in transgenic human 786-0 and murine ES cells. Based on prevailing models, Type 2B mutant pVHL proteins, including R167Q, are predicted to disrupt VBC complex formation by eliminating Elongin C binding to pVHL [13], [27]–[30]. We have previously observed the inability of R167Q HA-pVHL to bind Elongin C in a transgenic murine ES cell system [25].

**Figure 2:**
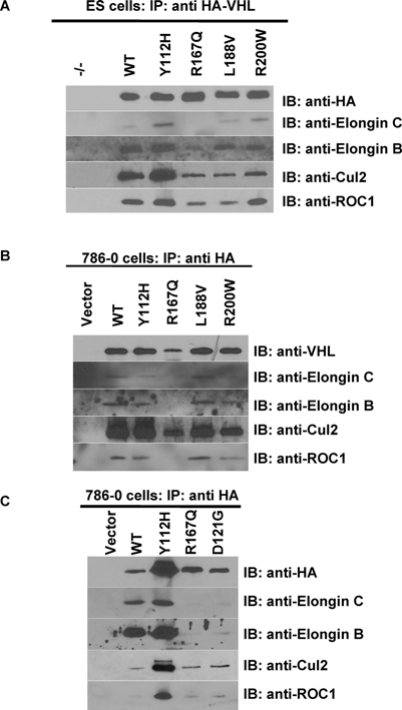
Co-immunoprecipitation of VBC complex proteins with type 2B mutant pVHL. A. Anti-HA immunoprecipitation of HA-pVHL and associated VBC complex members in transgenic ES cell clones. Anti-HA IP products were probed for successful pull-down of WT or mutant HA-pVHL and for co-IP of the indicated VBC complex members in stably-transfected *Vhl^−/−^* murine ES cells. B. and C. Anti-HA immunoprecipitation of HA-pVHL from transgenic 786-0 clones. Anti-HA IP products were probed for successful pull-down of WT or mutant HA-pVHL by anti-pVHL (B) or anti-HA (C) immunoblot and for co-IP of the indicated VBC complex members in stably-transfected 786-0 cells.

VBC complex formation was assessed by co-IP analysis of proteins interacting with HA-pVHL in *Vhl*-null murine ES cells expressing wild-type pVHL (WT), mutants Y112H, R167Q, L188V, and R200W, or no transgene (−/−). HA-pVHL pull-down was confirmed in each IP by anti-HA immunoblot (HA, Figure 2A). Each HA-pVHL-containing complex was then individually tested for interaction with known members of the VBC complex through protein-specific co-IP immunoblot (Figure 2A). As expected based on both previous reports and the localization of the respective mutations [20], [25], the R167Q HA-pVHL failed to substantially co-immunoprecipitate Elongin C, whereas wild-type HA-pVHL and mutant HA-pVHL representing Y112H, L188V, and R200W retained this interaction. Furthermore, wild-type HA-pVHL and mutant HA-pVHL representing Y112H, L188V, and R200W demonstrated interaction with complete VBC complex detecting the presence of murine Elongin B, Cul2, and Rbx1. Elongin B, Cul2, and Rbx1 also clearly associated with the R167Q HA-pVHL, suggesting that the VBC complex is at least partially intact in cells expressing this representative Type 2B *VHL* mutation.

In order to discern if the observed remnant VBC complex in R167Q HA-pVHL-expressing murine ES cells was an artifact of human-mouse interactions, we examined the same panel of *VHL* mutations for VBC complex formation in stably-transfected human 786-0 RCC cells. A second representative Type 2B *VHL* mutation D121G was included in this analysis to determine whether the formation of a remnant complex is limited to the specific Type 2B mutant R167Q HA-pVHL or is more broadly relevant to Type 2B VHL disease. Again, pVHL-associated proteins were co-immunoprecipitated, and pull-down of HA-pVHL was confirmed for each IP by anti-VHL (Figure 2B) or anti-HA (Figure 2C) immunoblot, followed by detection of known VBC complex members by protein-specific co-IP immunoblot (Figures 2B and 2C). Both R167Q and D121G mutant HA-pVHL failed to demonstrate a strong interaction with Elongin C, although robust association was detected for wild-type and the other mutant HA-pVHL proteins. The remaining VBC complex members were again associated with wild-type and all of the HA-pVHL mutants tested, including the Type 2B mutants R167Q and D121G. This experiment suggests that both of the Type 2B mutant HA-pVHL proteins studied may retain at least partial interaction with Elongin C and recruit a complex containing the essential human VBC E3 ubiquitin ligase components CUL2 and ROC1.

**Figure 3:**
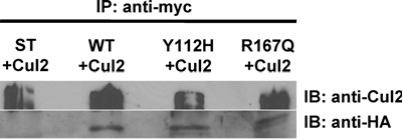
Reverse co-immunoprecipitation confirms CUL2-pVHL protein interaction in R167Q mutation. Anti-myc immunoprecipitation of myc-CUL2 and associated HA-pVHL in 786-0 clones. Stable 786-0 clones expressing vector-only (ST) or WT or mutant HA-pVHL were transiently transfected with wild-type myc-tagged CUL2 and subjected to myc-IP. Upper panel, IP of Myc-tagged Cul2 detected by anti-CUL2 immunoblot. Lower panel, co-IP of WT and mutant HA-pVHL detected by anti-HA immunoblot.

To confirm the observed interaction between R167Q HA-pVHL and CUL2, a myc-tagged CUL2 protein was transiently expressed in stable 786-0 cell lines expressing wild-type or RCC-associated mutant HA-pVHL for reverse co-IP studies (Figure 3). Cell extracts were subjected to anti-myc-agarose IP, and pull-down of myc-CUL2 was confirmed by anti-CUL2 immunoblot. Anti-HA co-IP immunoblot verified the results depicted in Figure 2B–C, displaying myc-CUL2 interaction with wild-type, Y112H, and R167Q HA-pVHL.

### Type 2B mutant pVHL retains HIF-1α-ubiquitylating activity

To determine if the remnant R167Q HA-pVHL–CUL2 complex retained E3 ubiquitin ligase activity, wild-type and mutant HA-pVHL were analyzed for competence to ubiquitinate HIF-1α using a modified version of the *in vitro* assay developed by Cockman *et al.* [18]. Exogenous HIF-1α was used as the ubiquitylation target in this experiment as the 786-0 parental cell line lacks confounding endogenous HIF-1α expression. The R167Q and a second Type 2B mutation (Q195X) have been shown to retain interaction with HIF-1α [20]. Retention of E3 ligase activity by the remnant R167Q mutant pVHL-CUL2 VBC complex, therefore, should correspond to preserved HIF-1α ubiquitylation *in vitro*.

**Figure 4:**
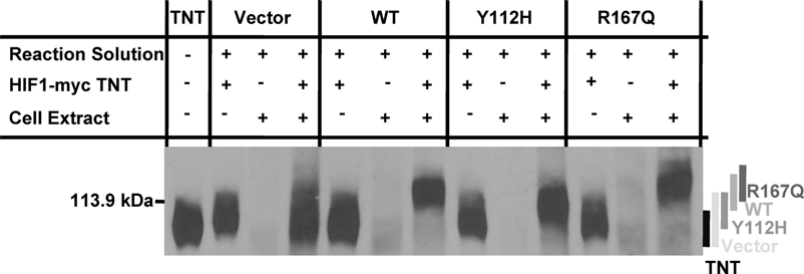
pVHL-dependent HIF-1α ubiquitylation in 786-0 cell lines. Anti-myc immunoblot to visualize migration shift of myc-tagged HIF-1α *in vitro*. Cell extracts from 786-0 clones expressing vector alone or WT or mutant HA-pVHL were incubated in the presence or absence of ubiquitylation reaction solution and TNT HIF-1α-myc and subjected to anti-myc immunoblot. A band representing unmodified TNT HIF-1α-myc, as depicted in the TNT lane, runs just below the ladder marker at 113.9 kDa. Upward shift of myc-HIF-1α, as seen in complete reaction lanes for WT and R167Q, indicates poly-ubiquitylation. The TNT HIF-1α-myc migration pattern for each complete reaction lane is graphically summarized to the right of the immunoblot.

*In vitro*-transcribed (TNT) myc-tagged full-length wild type human HIF-1α (HIF-1α-myc) was subjected to a modified *in vitro* ubiquitylation assay, using anti-myc immunoblot to visualize ubiquitylation of the HIF-1α-myc substrate (Figure 4, components of each analysis indicated above lane). The HIF-1α-myc protein migration for each cell line is summarized by a shaded line to the right of the immunoblot. TNT HIF-1α-myc substrate in the absence of reaction solution or cell extract served as a negative control for the unmodified electrophoretic mobility of the HIF-1α-myc protein. For each cell line in our assay, further controls were provided by excluding cell extract (first reaction) or TNT HIF-1α-myc substrate (second reaction). The third reaction for each cell line contained all three necessary components for the ubiquitylation reaction. An upward shift in HIF1α-myc mobility, as exemplified by the wild-type (WT) complete reaction, indicates HIF-1α-myc poly-ubiquitylation. The HIF-1α-myc protein was shifted only slightly upwards in the reaction utilizing extract from control vector-only 786-0 cells, demonstrating the basal pVHL-independent HIF-1α ubiquitylation present in this system. Type 2A Y112H mutant HA-pVHL failed to promote HIF-1α-myc mobility shift beyond basal levels, confirming previous work by Cockman *et al*. [18]. Notably, Type 2B R167Q mutant HA-pVHL promoted HIF-1α-myc mobility shift similar to WT HA-pVHL, demonstrating that the remnant R167Q HA-pVHL–Cullin-2 complex retains E3 ligase activity towards HIF-1α *in vitro*.

## Discussion

In addition to the strong association of sporadic ccRCC with biallelic *VHL* inactivation and of VHL disease-associated ccRCC with subtype-specific germline *VHL* mutations, evidence from xenograft models of tumor growth strongly supports the requirement for pVHL-mediated HIF regulation in suppression of renal tumorigenesis. However, several lines of evidence suggest that dose-dependent effects on basal HIF levels influence *VHL*-associated tumor development and behavior. Our prior investigation in eupoloid primary ES cell lines, utilized as a strategy to avoid interference from transforming cancer cell events, demonstrated a bias toward HIF-2α dysregulation for VHL Type 2B mutation, and a graduated degree of HIF dysregulation across the disease subtypes [25]. *In vitro* studies of RCC-predisposing Type 2A and Type 2B *VHL* missense mutations have revealed a correlation between the degree of mutant pVHL-mediated HIF-α dysregulation and risk of ccRCC [24]. In a recent study performed in both 786-0 and a second RCC-derived cell line, RCC4, representative Type 2A and Type 2B *VHL* mutations demonstrated intermediate levels of HIF stabilization [21]. Taken together, these results suggest that Type 2A and Type 2B mutant pVHL proteins retain an intermediate degree of HIF regulation, rather than an “all-or-none” pattern of regulation, likely contributing to the distinct phenotypes observed in these VHL disease subtypes.

In this study, we observed intermediate HIF regulatory activity by Type 2A Y112H and Type 2B R167Q mutant HA-pVHL, which could underlie the distinct genotype-phenotype correlations and may provide insight into the biology of sporadic RCC as well. In previous reports of VBC complex formation, the ability of disease-specific mutant pVHL to bind Elongin C was used as a proxy for ability to recruit the remainder of the VBC complex. The absence of 2B mutant HA-pVHL interaction with Elongin C in co-IP studies led to conclusions that α-domain mutations in pVHL abolish VBC complex formation [13], [27]–[30]. We report here, however, that both R167Q and D121G Type 2B mutant HA-pVHL participate in a complex with CUL2, ROC1, and Elongin B, as well as potentially with Elongin C either transiently or with greatly reduced abundance.

The co-immunoprecipitation of VBC complex members with R167Q and D121G mutant HA-pVHL could be due to reduced or transient formation of a wild-type VBC complex and/or formation of an alternate pVHL-Cullin-2 complex with ubiquitin ligase activity. The low abundance of Elongins C and B in association with both representative Type 2B mutant pVHL proteins may represent a limited quantity of stable complete VBC complex. Existing crystal structures depict the pVHL α-domain interacting with the VBC complex only through Elongin C [15]. However, the relatively abundant CUL2 and ROC1 in the mutant 2B VBC complex could point to a direct interaction between mutant HA-pVHL and CUL2 or the replacement of Elongins C and B with alternate adaptors linking the mutant HA-pVHL to CUL2–ROC1. Silver stain and proteomic analysis of our Type 2B mutant HA-pVHL immunoprecipitates failed to detect additional bands that could function as replacement adaptors in an alternate HA-pVHL–CUL2 complex (data not shown).

The Type 2B mutation R167Q has been shown to permit interaction between pVHL and HIF-α [20]. Therefore, if able to recruit an active complete or alternate VBC complex, Type 2B pVHL should be able to direct HIF-α ubiquitylation. Indeed, we observed that R167Q mutant HA-pVHL existed in complex with CUL 2 and ROC1 and mediated wild-type levels of HIF-1α poly-ubiquitylation *in vitro*. Though the presence of ROC1 has been shown to stabilize the VBC complex [12], our results cannot discern whether endogenous levels of Type 2B mutant pVHL expression support formation of a stable or transient complex with CUL2. R167Q mutant pVHL has been shown to be relatively unstable *in vitro* [24], and subtype-specific clinical manifestations of VHL disease may derive from a combination of mutant pVHL stability and the stability and activity of the mutant VBC complex.

In summary, we have demonstrated that disease-associated mutant pVHL proteins retain endogenous HIF-2α regulation. Two representative Type 2B mutant pVHL proteins partially preserved interaction with VBC complex members despite reduced binding to Elongin C, and the Type 2B mutant R167Q pVHL retained wild-type levels of ubiquitin ligase activity towards its target HIF-1α *in vitro*. Taken together, our results show that at least a subset of Type 2B *VHL* missense mutations result in a partial or unstable but active VBC complex which retains the ability to regulate HIF-α levels. Furthermore, our results support observations that *VHL* missense mutations generally confer lower levels of HIF-α stabilization than null or truncating Type 1 *VHL* mutations [21], [25] and provide mechanistic insight into this retained ubiquitin ligase activity.
